# Efficacy of adjuvant chemotherapy stratified by age and the 21-gene recurrence score in estrogen receptor-positive breast cancer

**DOI:** 10.1186/s12885-021-08461-9

**Published:** 2021-06-15

**Authors:** Jing Yu, Caijin Lin, Jiahui Huang, Jin Hong, Weiqi Gao, Siji Zhu, Lin Lin, Xiaosong Chen, Ou Huang, Jianrong He, Li Zhu, Weiguo Chen, Yafen Li, Jiayi Wu, Kunwei Shen

**Affiliations:** Department of General Surgery, Comprehensive Breast Health Center, Ruijin Hospital, Shanghai Jiao Tong University School of Medicine, Shanghai, 200025 China

**Keywords:** Breast cancer, 21-gene recurrence score, Chemotherapy, Age, Prognosis and prediction

## Abstract

**Background:**

The 21-gene recurrence score (RS) can predict chemotherapy benefit in estrogen receptor-positive, human epidermal growth factor receptor-2-negative (ER+/HER2-) early breast cancer patients. Age would influence the interaction between RS and chemotherapy effect. The current study aimed to determine RS thresholds which were predictive of chemotherapy benefit in young and old women, respectively.

**Methods:**

Patients diagnosed with pN0–1, ER+/HER2- breast cancer between 2009 and 2016 were retrospectively reviewed. Propensity score matching was performed according to chemotherapy usage. After stratifying patients with different cutoffs of age, the RS threshold indicating chemotherapy benefit in each age strata were determined by cox proportional hazard models.

**Results:**

A total of 1227 patients were included. The median age was 58 years and the median RS was 24. After matching, the RS thresholds suggesting chemotherapy benefit varied with age. For patients ≤55 years, chemotherapy benefit was observed in those having RS > 25 (*P* = 0.03), with 4-year invasive disease-free survival (IDFS) of 97.0 and 89.3% in patients receiving chemotherapy or not. While patients derived no benefit from chemotherapy if they had RS ≤25 (*P* = 0.66, 4-year IDFS: 95.3% vs. 94.6%). For patients > 55 years, adjuvant chemotherapy was associated with better prognosis in those with RS > 36 (*P* = 0.014, 4-year IDFS: 94.7% vs. 76.2%), but not in those having RS ≤36 (*P* = 0.13, 4-year IDFS: 92.3% vs. 95.8%).

**Conclusions:**

Old patients need higher RS thresholds to demonstrate the chemotherapy benefit. Further efforts are warranted to investigate the association between age and predictive RS thresholds.

**Supplementary Information:**

The online version contains supplementary material available at 10.1186/s12885-021-08461-9.

## Background

Breast cancer is biologically heterogeneous. Multigene assays for specific molecular subtype trace the intrinsic characteristics of tumors and contribute to risk assessment [1]. Among those assays, the 21-gene recurrence score (RS) testing is well-acknowledged and increasingly used for estrogen receptor-positive, human epidermal growth factor receptor-2-negative (ER+/HER2-) breast cancer patients [2].

The 21-gene RS is measured by the quantitative reverse transcriptase-polymerase chain reaction (RT-PCR) method, and classified as low-risk (< 18), intermediate-risk (18–30), and high-risk (> 30). It could quantify the risk of distant relapse as well as predict chemotherapy benefit in ER+/HER2-, lymph node (LN)-negative patients [3, 4]. The SWOG 8814 study then broadened the range of application to 1–3 LN-positive population [5]. The TAILORx and WSG Plan-B prospective trial used different cutoff values for risk stratification and consolidated the role of this genomic tool in guiding chemotherapy usage [6, 7].

Despite the multigene assay was established regardless of age, the difference in tumor biology and clinical features lead to distinct conditions between young and old. As shown in the subsequent report of the TAILORx trial, patients with midrange RS (11–25) derived no significant benefit from adjuvant chemotherapy. Whereas, subset analysis indicated that young patients (≤50 years) with RS 16–25 had a lower rate of distant recurrence with chemo-endocrine therapy [8]. Meanwhile, a post-hoc analysis of MINDACT also showed different absolute chemotherapy benefit in young versus old [9]. Those all indicate that age has an effect on the interaction between genomic risk and chemotherapy efficacy.

At present, the 21-gene RS is widely recommended by international guidelines [10, 11]. However, when taking age into account, the clear bound of RS to demonstrate chemotherapy benefit had only been evaluated in young patients (≤ 50 years) but not in older women. Meanwhile, most of the conclusions were drawn from the western population, and there were limited data on the prognostic and predictive value of RS in Asians. In the current study, we included a large cohort of Chinese patients and aim to figure out the specific RS threshold which can predict the chemotherapy benefit in young and old patients, respectively.

## Methods

### Study population and follow up information

Patients diagnosed with invasive breast cancer and underwent surgical treatment between January 2009 and December 2016 at Shanghai Ruijin hospital were retrospectively reviewed. Data on clinicopathological parameters including age, menopausal status, tumor size, lymph node status, histology, and tumor grade, treatment, and follow-up information, were retrieved from prospectively-maintained Shanghai Jiao Tong University Breast Cancer Database (SJTU-BCDB). The study was limited to ER+/HER2- breast cancer patients with 0–3 axillary lymph nodes involved. Exclusion criteria were: 1) de novo stage IV disease; 2) patients who had received neoadjuvant treatment; 3) patients with incomplete data of baseline characteristic or follow-up information; 4) patients without available reports of the 21-gene test. Clinical end points of interest included Invasive Disease-Free Survival (IDFS) and Distant Disease Free Survival (DDFS) according to the STEEP system [12]. The current study was approved by the Ethical Committees of Shanghai Ruijin Hospital.

### Pathological, immunohistochemical (IHC) analysis and the 21-gene RS assay

Pathological and IHC analysis was performed at the Department of Pathology, Ruijin Hospital. IHC staining was performed on 4-μm-thick FFPE tissue section with the following antibodies: ER (clone 1D5; 1:100; rabbit monoclonal; Dako), HER2 (clone 4B5, rabbit monoclonal; 1:100; Roche). ER positivity was defined as nuclear staining in ≥1% of tumor cells. HER2 was considered negative with 0–1 by IHC or negative by FISH. The 21-gene RS assay was performed on FFPE tissues as described in our previous study [13]. Total RNA was extracted from three 10-μm unstained sections using the RNeasy FFPE RNA kit (Qiagen, Germany) after identifying the absence of DNA contamination. Gene-specific reverse transcription was performed on the Omniscript RT kit (Qiagen, Germany) followed by standardized qPCR with Premix Ex TaqTM (Takara Bio, Inc.) in Applied Biosystems 7500 Real-Time PCR system (Foster City, CA). The expression levels of 16 cancer-related genes were measured in triplicate and normalized by 5 reference genes. The RS, ranging from 0 to 100, was then calculated by using a specific algorithm [3].

### Statistical analysis

Baseline characteristics were compared between patients receiving adjuvant chemotherapy or not by the Chi-square test. The prognostic value of RS was evaluated by using the Kaplan-Meier method and compared using the log-rank test. When analyzing the chemotherapy benefit, a one-to-one propensity score matching (PSM) was performed between women receiving chemotherapy or not using the nearest-neighbor matching method with a caliper distance of 0.3 [14]. Cox proportional hazard models were used to judge if the RS value was predictive of chemotherapy benefit. This was accomplished by determining the statistical significance of the interaction term with the likelihood ratio test in a model that included chemotherapy, the RS groups, and the interaction between chemotherapy and RS groups. We then stratified the patients according to different ages (range, 30–65 years). In each age group, interaction analysis as above mentioned was performed for each specific RS cutoff (range, 5–40). Two-sided *P* value ≤0.05 was considered statistically significant. Analyses were performed using R software (version 3.6.3; www.r-project.org).

## Results

### Baseline characteristics before and after PSM

A total of 1227 patients were included eventually. The median RS was 24 (interquartile range, 17–31). There were 311 (25.3%) patients having RS of 0–17, 586 (47.8%) having RS of 18–30, and 330 (26.9%) having RS ≥31, respectively. Baseline characteristics were presented in Table 1. The median age of patients was 58 years (interquartile range, 44 to 66) and 436 (35.5%) of patients were premenopausal. Only 71 (5.8%) had 1–3 lymph nodes involvement. A total of 477 (38.9%) patients received tamoxifen-containing adjuvant endocrine therapy, and 750 (61.1%) patients received aromatase inhibitors. Ovarian function suppression (OFS) was administrated in 51 patients.

**Table 1:** Base characteristics of patients before and after propensity score matching

	Before propensity score matching ***n*** = 1227				After propensity score matching ***n*** = 700			
	Total population N (%)	Chemotherapy N (%)	No Chemotherapy N (%)	*P*	Total population N (%)	Chemotherapy N (%)	No Chemotherapy N (%)	*P*
Age				<.001				.065
< 50	369 (30.1)	218 (35.3)	151 (24.8)		198 (28.3)	88 (25.1)	110 (31.4)	
≥50	858 (69.9)	399 (64.7)	459 (75.2)		502 (71.7)	262 (74.9)	240 (68.6)	
Menopausal status				<.001				.111
Pre-	436 (35.5)	256 (41.5)	180 (29.5)		240 (34.3)	110 (31.4)	130 (37.1)	
Post-	791 (64.5)	361 (58.5)	430 (70.5)		460 (65.7)	240 (68.6)	220 (62.9)	
Tumor size, cm				<.001				.621
≤2	843 (68.7)	384 (62.2)	459 (75.2)		490 (70.0)	242 (69.1)	248 (70.9)	
> 2	384 (31.3)	233 (37.8)	151 (24.8)		210 (30.0)	108 (30.9)	102 (29.1)	
Node involvement				<.001				.135
Negative	1156 (94.2)	557 (90.3)	599 (98.2)		670 (95.7)	331 (94.6)	339 (96.9)	
Positive	71 (5.8)	60 (9.7%)	11 (1.8)		30 (4.3)	19 (5.4)	11 (3.1)	
Histology				<.001				.489
IDC	1059 (86.3)	582 (94.3)	477 (78.2)		629 (89.9)	319 (91.1)	310 (88.6)	
ILC	50 (4.1)	21 (3.4)	29 (4.8)		37 (5.3)	17 (4.9)	20 (5.7)	
Other	118 (9.6)	14 (2.3)	104 (17.0)		34 (4.8)	14 (4.0)	20 (5.7)	
Grade				<.001				.171
Well	116 (9.4)	26 (4.2)	90 (14.8)		46 (6.6)	25 (7.1)	21 (6.0)	
Moderately	683 (55.7)	342 (55.4)	341 (55.9)		463 (66.1)	229 (65.4)	234 (66.9)	
Poorly	264 (21.5)	212 (34.4)	52 (8.5)		119 (17.0)	67 (19.1)	52 (14.9)	
Unknown	164 (13.4)	37 (6.0)	127 (20.8)		72 (10.3)	29 (8.3)	43 (12.3)	
Endocrine therapy				.002				.211
TAM	477 (38.9)	266 (43.1)	211 (34.6)		262 (37.4)	123 (35.1)	139 (39.7)	
AI	750 (61.1)	351 (56.9)	399 (65.4)		438 (62.6)	227 (64.9)	211 (60.3)	

There were 617 (50.3%) out of 1227 patients having adjuvant chemotherapy. Patients who received chemotherapy were more likely to be younger (35.3% vs. 24.8%, *P* < 0.001), have larger tumors (37.8% vs. 24.8%, *P* < 0.001), have histology of IDC (94.3% vs. 78.2%, *P* < 0.001), and have poorly differentiated tumors (34.4% vs. 8.5%, *P* < 0.001). As expected, more patients diagnosed with the node-positive disease in the chemotherapy-treated group (9.7% vs. 1.8%, *P* < 0.001). Those who received chemotherapy also showed a trend for receiving tamoxifen (43.1% vs. 34.6%, *P* = 0.002).

**Fig. 1:**
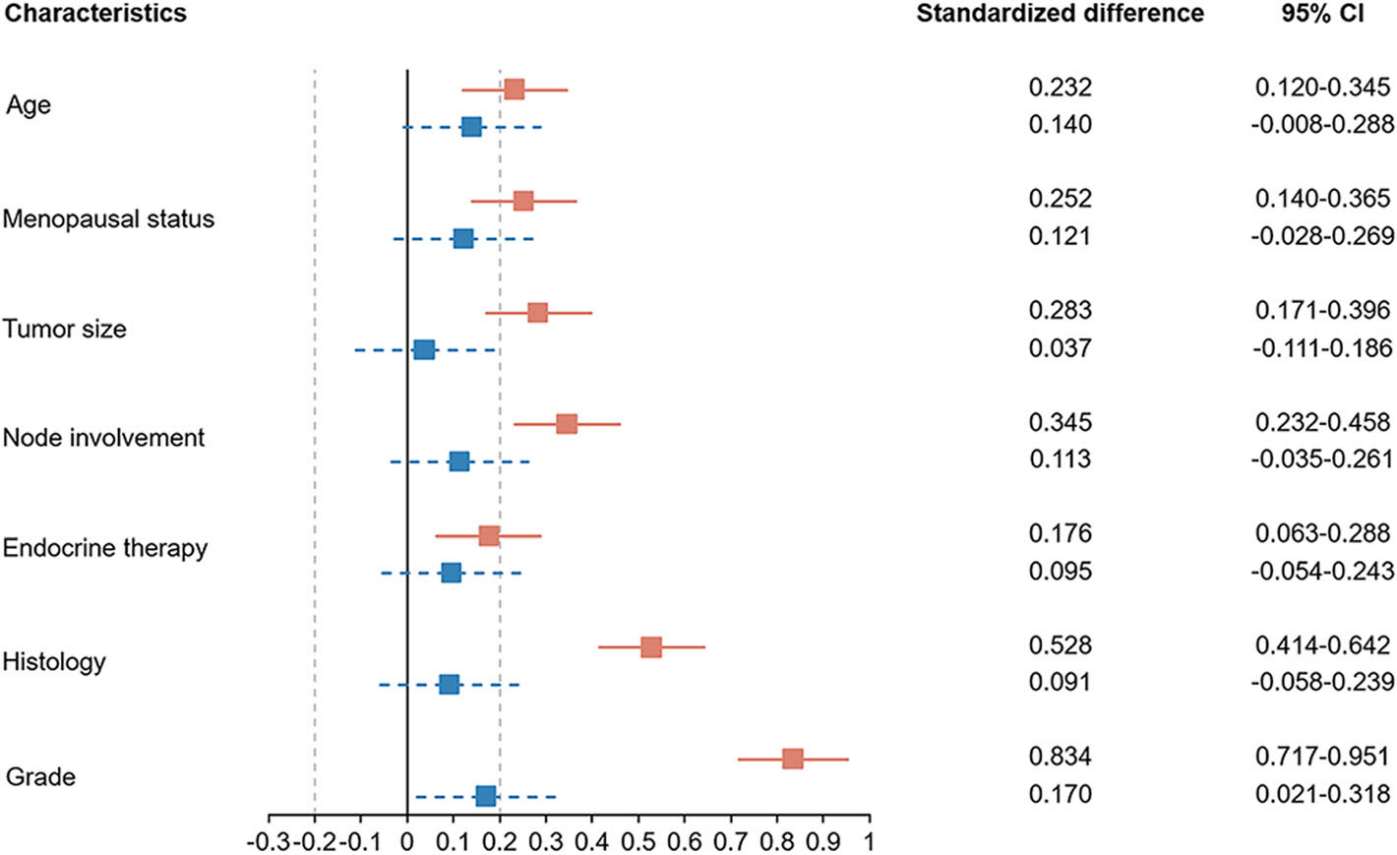
Adjustment for imbalances in baseline covariates by propensity score matching. The red squares represent the standard difference of propensity score between chemotherapy and no-chemotherapy groups before PSM. The blue squares represent the standard difference of propensity score between two groups after PSM. The standardized difference for all covariates met the criteria of < 20% after PSM

Variables significantly differed between patients receiving chemotherapy or not were put into the PSM algorithm. After PSM, the chemotherapy and no chemotherapy group consisted of 350 patients, respectively. As shown in Table 1, clinicopathological characteristics were well-balanced after matching. The distribution patterns of the propensity score were similar between patients with or without chemotherapy (Figure S1), and the standardized difference for all covariates met the criteria of < 20% (Fig. 1).

### Recurrence score and prognosis

**Fig. 2:**
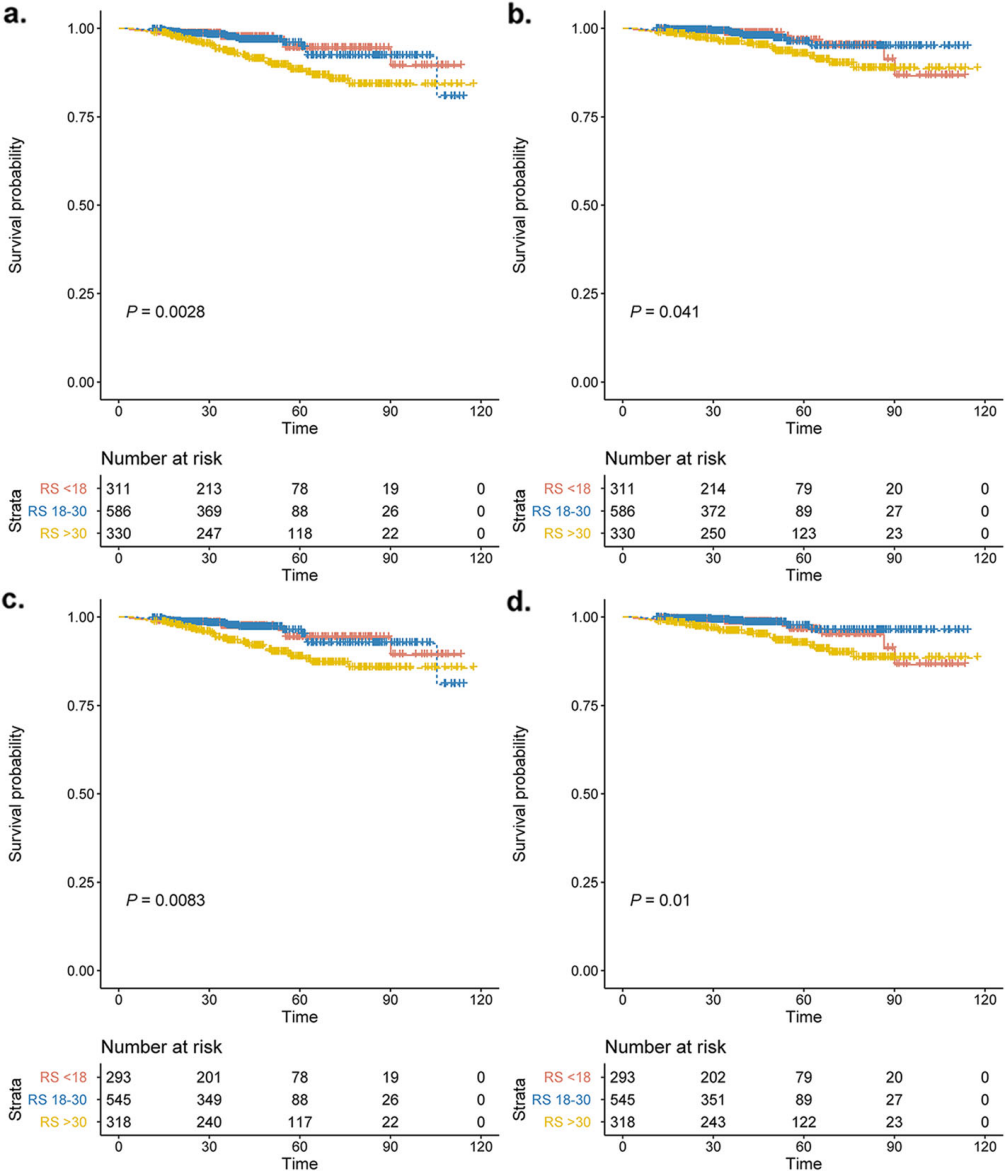
Kaplan-Meier estimates for IDFS and DDFS according to RS group. Rate of Invasive disease-free survival (**a**) and Distant disease-free survival (**b**) for the entire cohort. Rate of Invasive disease-free survival (**c**) and Distant disease-free survival (**d**) for the lymph node-negative cohort. The box under each graph presents the number of patients at risk at each time point

The median follow-up was 41.7 months (interquartile range, 18.1 to 93.1 months). A total of 87 events were observed, including 18 invasive local/regional recurrences, 13 contralateral breast cancers, 21 distant metastases, 20 s primary invasive cancers, and 15 deaths. The Kaplan-Meier estimates for IDFS and DDFS were shown in Fig. 2. The categorical RS was significantly prognostic for IDFS (*P* = 0.0028; Fig. 2A) and DDFS (*P* = 0.041; Fig. 2B) in overall patients, as well as in the 1156 lymph node-negative patients (IDFS, *P* = 0.0083; Fig. 2C; and DDFS, *P* = 0.01; Fig. 2D). Meanwhile, when analyzed as continuous variable, univariate analysis also demonstrated that RS was a significant predictor for IDFS (HR = 1.013, 95% CI 1.002–1.025, *P* = 0.024) and DDFS (HR = 1.025, 95% CI 1.010–1.040, *P* = 0.001).

### Predictive value of RS on chemotherapy and in patients of different ages after PSM

The efficacy of chemotherapy was evaluated in the matched cohort. Overall, the interactions between chemotherapy and RS groups for IDFS were obvious when RS ≥31, and there was a trend for interaction (*P* < 0.1) when RS = 26 (Figure S2), which was consistent with previous studies.

**Fig. 3:**
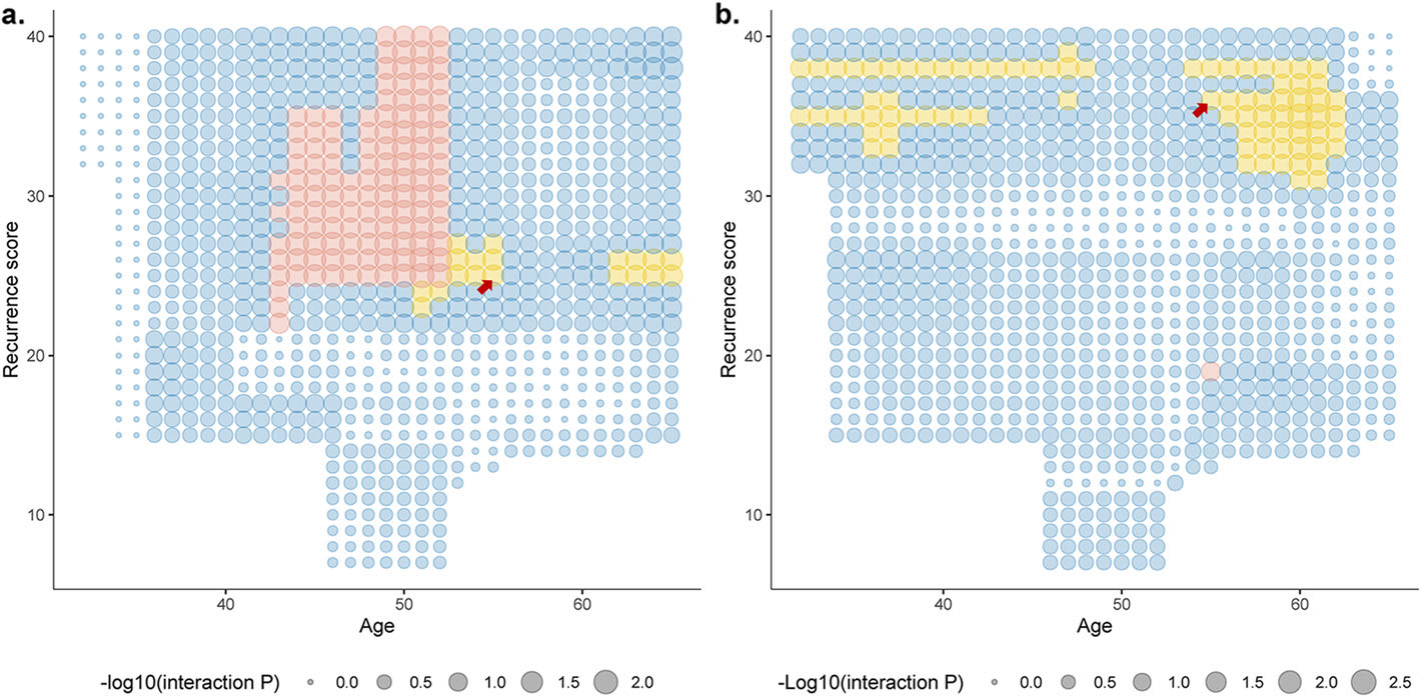
Interaction between different RS cutoffs and chemotherapy in young and old patients. The size of the point in Figure 3 represents the negative logarithm of interaction *P*-value. The bigger the point, the less the *P*-value, which means the higher the statistical significance. Blue points represent the interaction *P*-value ≥0.05. The red points represent that although the *P*-value < 0.05, the 95% confidence interval of the hazard ratio for chemotherapy ranges 0 to infinite, due to the few numbers of patients or events. Only the yellow points represent the valid RS thresholds, which have significant interaction with chemotherapy usage. The selection of RS thresholds followed this process: 1) Selecting an age, which has valid RS values (yellow points) both in **A** and **B**. 2) Choosing the RS threshold with the highest statistical significance, which is 25 in **A** and 36 in **B** (indicated by the arrow)

Then patients were stratified by different cutoffs of age, and RS thresholds suggesting chemotherapy benefit were identified separately in young (≤age cutoff; Fig. 3A) and old (>age cutoff; Fig. 3B) cohorts. As was shown, the RS thresholds with predictive value were different across age groups. When setting 55 years as the age cutoff, predictive RS thresholds could be observed in both cohorts. We selected the RS value that reached the highest statistical significance in manifesting the interaction with chemotherapy, which was 25 in the young cohort and 36 in the old cohort.

**Fig. 4:**
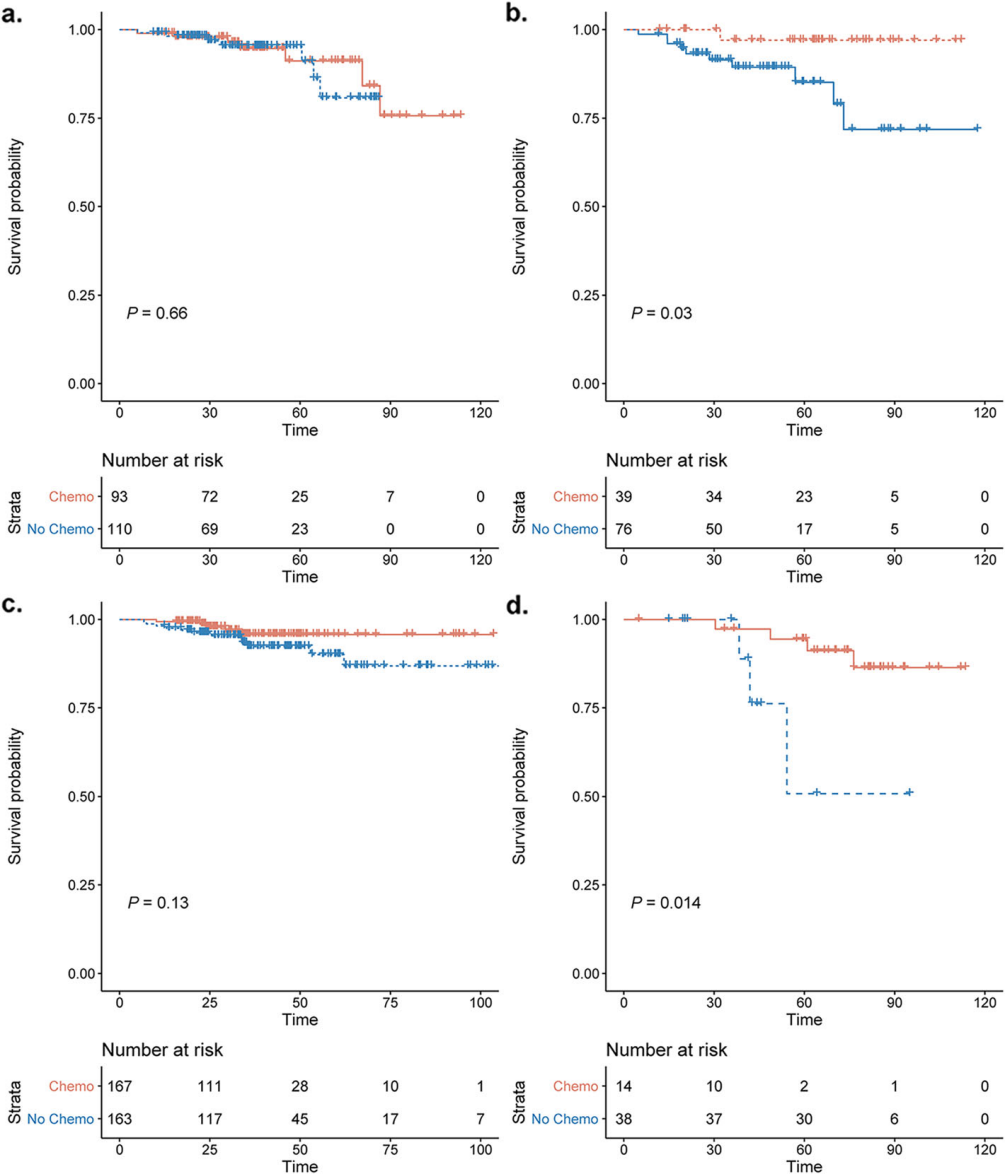
Prognosis of patients according to chemotherapy usage at 55 years of age. Rates of Invasive disease-free survival according to chemotherapy usage in (**a**) patients ≤55 years with RS ≤25, (**b**) patients ≤55 years with RS > 25, (**c**) patients > 55 years with RS ≤36, and (**d**) patients > 55 years with RS > 36

IDFS of patients according to chemotherapy usage were compared in specific strata defined by age and RS. Kaplan-Meier curves illustrated that patients with age ≤ 55 years and RS ≤25 derived no benefit from additional chemotherapy (*P* = 0.66; Fig. 4A), and 4-year invasive disease-free survival (IDFS) was 95.3 and 94.6% in patients receiving chemotherapy or not. Whereas significant benefit of chemotherapy was observed in young patients who had RS > 25 (*P* = 0.03; Fig. 4B), with 4-year IDFS of 97.0 and 89.3%, respectively. Furthermore, for patients older than 55 years, adjuvant chemotherapy was associated with better prognosis in those who had RS > 36 (*P* = 0.014; Fig. 4D), with 4-year IDFS of 94.7 and 76.2% respectively. No apparent benefit was seen in those older than 55 years and having RS ≤36 (*P* = 0.13, 4-year IDFS: 92.3% vs. 95.8%; Fig. 4C; Table 2).

**Table 2:** Survival of breast cancer patients receiving adjuvant chemotherapy or not stratified by age and the 21 gene Recurrence Score

	Chemoendocrine therapy		Endocrine therapy		*P*-value
	4-year IDFS rate (%)	95% CI (%)	4-year IDFS rate (%)	95% CI (%)	
≤55 years					
RS ≤25	95.3	90.7–100.0	94.6	89.6–100.0	0.66
RS > 25	97.0	91.3–100.0	89.3	82.0–97.3	0.03
> 55 years					
RS ≤36	92.3	87.7–97.2	95.8	92.1–99.6	0.13
RS > 36	94.7	87.9–100.0	76.2	52.1–100.0	0.014

## Discussion

In the current study, we demonstrated the distribution of RS in a cohort of 1227 Chinese breast cancer patients and validated the prognostic value of RS. Notably, the RS thresholds that can indicate the chemotherapy benefit varied with age. For example, young patients (≤55 years) derived no apparent benefit of chemotherapy unless they had RS > 25, whereas old patients (> 55 years) could benefit from adjuvant chemotherapy when the RS was > 36.

Regarding the distribution of RS, data from Surveillance, Epidemiology, and End Results (SEER) showed that patients who had RS of 0–17, 18–30, and > 30 accounted for 56.52, 35.74, and 7.74% [15]. In Europe, the distribution was 55.2, 35.2, and 9.6%, respectively [16]. In our study, only 25.3% of patients had low-risk RS, and more patients were classified as intermediate- (47.7%) or high-risk RS (26.9%) than other studies. There may be two possible reasons. First, only 30% of eligible ER-positive patients in the United States and 20% in Europe received the 21-gene RS testing [16, 17]. For patients with clinical unfavorable characteristics, such as having lymph node involvement or poorly differentiated nuclear grade, physicians may find sufficient evidence in recommending chemotherapy directly. Hence, a considerable number of patients with a high risk of relapse forgo this testing, who were originally enriched for intermediate to high RS. However, all eligible patients consecutively underwent this multigene assay in our center, and our results may better reflect the real-world RS distribution. Another reason may be the racial disparity. Actually, the median age at diagnosis in China is nearly a decade younger than that in the United States, and advanced-stage breast cancer is more frequently presented [18]. Those indicate that breast cancer in China may be more aggressive than that in western countries. We further compared the RS distribution with other Asian cohorts. In a Japanese study, the three risk groups represented 48%, 20%, and 33% [19]. We noticed that the authors only enrolled patients who received tamoxifen but not chemotherapy, and their distribution was close to that in the NSABP B-14 trial. In another Chinese cohort by Yang et al., the distribution of three RS groups was 33.8, 45.5, and 20.7%, which is similar to our results [20].

The prognostic value of RS was initially validated in ER-positive lymph node-negative breast cancer patients who received tamoxifen. The NSABP B-20 further showed that RS can predict the difference in distant relapse rate, both in the chemotherapy-treated and untreated cohort [4]. Furthermore, the TransATAC study and the ECOG E2197 study demonstrated that RS was predictive for the recurrence risk, regardless of the nodal status [21, 22]. In the current study, we have analyzed patients’ survival according to RS stratification in the whole patients and the lymph node-negative patients. Consistent with the previous study, our results showed the robust prognostic value of RS, which would not be eliminated by the administration of chemotherapy or the lymph node status. Whereas, only 5.8% of the patients had 1–3 LN metastasis. This proportion would increase in the future, especially in postmenopausal patients, since the results of the prospective RxPONDER trial have just been released [23].

In TAILORx, there was an interaction between age and chemotherapy for disease outcomes [8]. In addition, Kizy et al. using SEER data showed that chemotherapy could bring about survival improvement in young but not older patients (≥70 years) with high-risk RS [24]. These suggested that the chemotherapy efficacy was different among young and old women. While, there was limited data on chemotherapy benefit, as being interpreted by RS, in old patients. In the current study, we also observed that the RS thresholds indicating chemotherapy benefit were different across ages. A plausible explanation was that for old patients, the benefit of chemotherapy is directly due to cytotoxic action, while young patients especially premenopausal women can also benefit from chemotherapy-induced ovarian suppression [25]. Indeed, in TAILORx, the chemotherapy benefit was most evident in premenopausal patients at 45–50 years, consistent with the effect of chemotherapy-induced-amenorrhea (CIA) [26]. Another reason is that the fragility and morbid condition may call for more tolerable but less effective regimens in old patients. Besides, several studies demonstrated that breast cancer of young patients was biologically more aggressive than that in old women [27, 28]. The intrinsic oncological difference between young and old may alter the performance of the 21-gene RS, as young patients tend to have a higher proportion of high-risk RS [29]. From the above, we prompt that adjuvant chemotherapy guided by the 21-gene RS should be discussed separately in young and old patients.

The NSABP B-20 showed that the absolute chemotherapy benefit seems to increase continuously with rising RS (up to 50), suggesting that there may be a cutoff point for RS to demonstrate the effect of chemotherapy [4]. A retrospective study using SEER data showed that this RS threshold was approximately 26 [30]. In the current study, RS of 26 also displayed a marginally significant interaction with chemotherapy. When taking age into account, we observed that the RS threshold to demonstrate chemotherapy benefit was 25 in patients ≤55 years. Liu et al. included 2721 patients younger than 40 years and found that the receipt of chemotherapy was associated with better breast cancer-specific survival in patients with RS > 25 (hazard ratio = 0.369, *P* = 0.028, [31]). And results of the Young Women’s Breast Cancer Study showed no survival difference in mid-range RS (11–25) patients receiving chemotherapy or not [32]. These indicated that RS of 25 tends to be a robust value that can reflect the chemotherapy benefit in young patients. Besides, approximately 34% of patients in the TAILORx were premenopausal, and only 12.5% of them received OFS. In the current study, the proportion were 35.5 and 11.7%, correspondingly. Since OFS could also address the risk of relapse [33, 34], the RS threshold to predict chemotherapy benefit in those patients warrant cautious interpretation.

On the other end of the spectrum, we found that the RS threshold indicating the chemotherapy benefit was 36 in patients older than 55 years, which has never been defined in previous studies. We postulate the higher RS threshold for chemotherapy in old patients was due to the less magnitude of benefit. Of note, determining this clear bound of RS is particularly necessary since elders were more likely to have ER-positive breast cancer and comprise the majority of patients who receive the 21-gene RS testing [35]. Meanwhile, conservative treatment-prescribing was always favored in them, because the toxicity and mortality due to chemotherapy could overweigh the absolute benefit of therapy. Our result suggests that old patients with an RS of 25 to 36 might avoid cytotoxicity from unnecessary chemotherapy, or at least could consider using less aggressive regimens.

The strength of this research was that we conducted the study in a population-based Chinese breast cancer series and defined the RS threshold for chemotherapy recommendation in young and old patients separately. Still, there were several limitations. First, selection bias may serve as an inevitable defect of the retrospective study, although we consecutively performed the 21-gene RS testing in eligible patients and used the propensity score matching to narrow the confounding effect. Second, the follow-up period is relatively short since the luminal breast cancer carries a consistent risk of recurrence for 15 years [36], whereas the effect of adjuvant chemotherapy was mostly seen in the first few years after surgery [37]. Last but not least, therapeutic efficacy due to different chemotherapy regimens as well as the administration of OFS was not considered in the current study, which deserves more detailed research.

## Conclusions

We investigate the prognostic and predictive value of 21-gene RS in a large cohort of Chinese luminal breast cancer patients with 0–3 lymph nodes involved. Meanwhile, we found that the RS value to demonstrate chemotherapy benefit was higher in old patients than in young ones. The relationship between age and predictive RS value warrants further evaluation.

## Supplementary Information

**Additional file 1.**

## Data Availability

The datasets generated during and/or analyzed during the current study are available from the corresponding author on reasonable request at http://bcdb.mdt.team:8080.

## Abbreviations

ER: Estrogen receptor
HER2: Human epidermal growth factor receptor-2-negative
RS: Recurrence score
RT-PCR: Reverse transcriptase-polymerase chain reaction
LN: Lymph node
IDFS: Invasive Disease-Free Survival
DDFS: Distant Disease-Free Survival
IHC: Immunohistochemical
PSM: Propensity score matching
IDC: Invasive ductal carcinoma
OFS: Ovarian function suppression
SEER: Surveillance, Epidemiology, and End Results
CIA: Chemotherapy-induced-amenorrhea

## References

[1] Kwa M, Makris A, Esteva FJ (2017). Clinical utility of gene-expression signatures in early stage breast cancer. Nat Rev Clin Oncol.

[2] Curtit E, Mansi L, Maisonnette-Escot Y, Sautière JL, Pivot X (2017). Prognostic and predictive indicators in early-stage breast cancer and the role of genomic profiling: focus on the Oncotype DX breast recurrence score assay. Eur J Surg Oncol.

[3] Paik S, Shak S, Tang G, Kim C, Baker J, Cronin M, Baehner FL, Walker MG, Watson D, Park T, Hiller W, Fisher ER, Wickerham DL, Bryant J, Wolmark N (2004). A multigene assay to predict recurrence of tamoxifen-treated, node-negative breast cancer. N Engl J Med.

[4] Paik S, Tang G, Shak S, Kim C, Baker J, Kim W, Cronin M, Baehner FL, Watson D, Bryant J, Costantino JP, Geyer CE, Wickerham DL, Wolmark N (2006). Gene expression and benefit of chemotherapy in women with node-negative, estrogen receptor-positive breast cancer. J Clin Oncol.

[5] Albain KS, Barlow WE, Shak S, Hortobagyi GN, Livingston RB, Yeh IT, Ravdin P, Bugarini R, Baehner FL, Davidson NE, Sledge GW, Winer EP, Hudis C, Ingle JN, Perez EA, Pritchard KI, Shepherd L, Gralow JR, Yoshizawa C, Allred DC, Osborne CK, Hayes DF, Breast Cancer Intergroup of North America (2010). Prognostic and predictive value of the 21-gene recurrence score assay in postmenopausal women with node-positive, oestrogen-receptor-positive breast cancer on chemotherapy: a retrospective analysis of a randomised trial. Lancet Oncol.

[6] Sparano JA, Gray RJ, Makower DF, Pritchard KI, Albain KS, Hayes DF, Geyer CE, Dees EC, Perez EA, Olson JA (2015). Prospective validation of a 21-gene expression assay in breast Cancer. N Engl J Med.

[7] Gluz O, Nitz UA, Christgen M, Kates RE, Shak S, Clemens M, Kraemer S, Aktas B, Kuemmel S, Reimer T, Kusche M, Heyl V, Lorenz-Salehi F, Just M, Hofmann D, Degenhardt T, Liedtke C, Svedman C, Wuerstlein R, Kreipe HH, Harbeck N (2016). West German study group phase III PlanB trial: first prospective outcome data for the 21-gene recurrence score assay and concordance of prognostic markers by central and local pathology assessment. J Clin Oncol.

[8] Sparano JA, Gray RJ, Makower DF, Pritchard KI, Albain KS, Hayes DF, Geyer CE, Dees EC, Goetz MP, Olson JA (2018). Adjuvant chemotherapy guided by a 21-gene expression assay in breast Cancer. N Engl J Med.

[9] Piccart MJ, Poncet C, Cardoso F, van't Veer L, Delaloge S, Pierga J-Y, Brain E, Vrijaldenhoven S, Neijenhuis P, Aalders K (2020). Abstract GS4-05: should age be integrated together with clinical and genomic risk for adjuvant chemotherapy decision in early luminal breast cancer? MINDACT results compared to those of TAILOR-X. AACR.

[10] Gradishar WJ, Anderson BO, Abraham J, Aft R, Agnese D, Allison KH, Blair SL, Burstein HJ, Dang C, Elias AD, Giordano SH, Goetz MP, Goldstein LJ, Isakoff SJ, Krishnamurthy J, Lyons J, Marcom PK, Matro J, Mayer IA, Moran MS, Mortimer J, O'Regan RM, Patel SA, Pierce LJ, Rugo HS, Sitapati A, Smith KL, Smith ML, Soliman H, Stringer-Reasor EM, Telli ML, Ward JH, Young JS, Burns JL, Kumar R (2020). Breast Cancer, version 3.2020, NCCN clinical practice guidelines in oncology. J Natl Compr Cancer Netw.

[11] Andre F, Ismaila N, Henry NL, Somerfield MR, Bast RC, Barlow W, Collyar DE, Hammond ME, Kuderer NM, Liu MC, van Poznak C, Wolff AC, Stearns V (2019). Use of biomarkers to guide decisions on adjuvant systemic therapy for women with early-stage invasive breast Cancer: ASCO clinical practice guideline update-integration of results from TAILORx. J Clin Oncol.

[12] Hudis CA, Barlow WE, Costantino JP, Gray RJ, Pritchard KI, Chapman J-AW, Sparano JA, Hunsberger S, Enos RA, Gelber RD, Zujewski JA (2007). Proposal for standardized definitions for efficacy end points in adjuvant breast cancer trials: the STEEP system. J Clin Oncol.

[13] Wu J, Fang Y, Lin L, Fei X, Gao W, Zhu S, Zong Y, Chen X, Huang O, He J (2017). Distribution patterns of 21-gene recurrence score in 980 Chinese estrogen receptor-positive, HER2-negative early breast cancer patients. Oncotarget.

[14] Austin PC (2011). An introduction to propensity score methods for reducing the effects of confounding in observational studies. Multivariate Behav Res.

[15] Petkov VI, Miller DP, Howlader N, Gliner N, Howe W, Schussler N, Cronin K, Baehner FL, Cress R, Deapen D, Glaser SL, Hernandez BY, Lynch CF, Mueller L, Schwartz AG, Schwartz SM, Stroup A, Sweeney C, Tucker TC, Ward KC, Wiggins C, Wu XC, Penberthy L, Shak S (2016). Breast-cancer-specific mortality in patients treated based on the 21-gene assay: a SEER population-based study. NPJ Breast Cancer.

[16] Albanell J, Svedman C, Gligorov J, Holt SDH, Bertelli G, Blohmer J-U, Rouzier R, Lluch A, Eiermann W (2016). Pooled analysis of prospective European studies assessing the impact of using the 21-gene recurrence score assay on clinical decision making in women with oestrogen receptor-positive, human epidermal growth factor receptor 2-negative early-stage breast cancer. Eur J Cancer.

[17] Orucevic A, Heidel RE, Bell JL (2016). Utilization and impact of 21-gene recurrence score assay for breast cancer in clinical practice across the United States: lessons learned from the 2010 to 2012 National Cancer Data Base analysis. Breast Cancer Res Treat.

[18] Fan L, Strasser-Weippl K, Li J-J, St Louis J, Finkelstein DM, Yu K-D, Chen W-Q, Shao Z-M, Goss PE (2014). Breast cancer in China. Lancet Oncol.

[19] Toi M, Iwata H, Yamanaka T, Masuda N, Ohno S, Nakamura S, Nakayama T, Kashiwaba M, Kamigaki S, Kuroi K (2010). Clinical significance of the 21-gene signature (Oncotype DX) in hormone receptor-positive early stage primary breast cancer in the Japanese population. Cancer.

[20] Yu-Qing Y, Lei W, Mei-Ling H, Jing-Jing X, Mei-Chen W, Jiang W, Jun-Sheng H, Rui L, Nan-Lin L (2019). Clinical significance of 21-gene recurrence score assay for hormone receptor-positive, lymph node-negative breast cancer in early stage. Exp Mol Pathol.

[21] Dowsett M, Cuzick J, Wale C, Forbes J, Mallon EA, Salter J, Quinn E, Dunbier A, Baum M, Buzdar A, Howell A, Bugarini R, Baehner FL, Shak S (2010). Prediction of risk of distant recurrence using the 21-gene recurrence score in node-negative and node-positive postmenopausal patients with breast cancer treated with anastrozole or tamoxifen: a TransATAC study. J Clin Oncol.

[22] Goldstein LJ, Gray R, Badve S, Childs BH, Yoshizawa C, Rowley S, Shak S, Baehner FL, Ravdin PM, Davidson NE, Sledge GW, Perez EA, Shulman LN, Martino S, Sparano JA (2008). Prognostic utility of the 21-gene assay in hormone receptor-positive operable breast cancer compared with classical clinicopathologic features. J Clin Oncol.

[23] Kalinsky K, Barlow WE, Meric-Bernstam F, Gralow JR, Albain KS, Hayes D, Lin N, Perez EA, Goldstein LJ, Chia S: Abstract GS3-00: first results from a phase III randomized clinical trial of standard adjuvant endocrine therapy (ET)+/−chemotherapy (CT) in patients (pts) with 1-3 positive nodes, hormone receptor-positive (HR+) and HER2-negative (HER2-) breast cancer (BC) with recurrence score (RS)< 25: SWOG S1007 (RxPonder)**.** In: AACR; 2021.

[24] Kizy S, Altman AM, Marmor S, Denbo JW, Jensen EH, Tuttle TM, Hui JYC (2019). 21-gene recurrence score testing in the older population with estrogen receptor-positive breast cancer. J Geriatr Oncol.

[25] Walshe JM, Denduluri N, Swain SM (2006). Amenorrhea in premenopausal women after adjuvant chemotherapy for breast cancer. J Clin Oncol.

[26] Sparano JA, Gray RJ, Ravdin PM, Makower DF, Pritchard KI, Albain KS, Hayes DF, Geyer CE, Dees EC, Goetz MP (2019). Clinical and genomic risk to guide the use of adjuvant therapy for breast Cancer. N Engl J Med.

[27] Chen H-L, Zhou M-Q, Tian W, Meng K-X, He H-F (2016). Effect of age on breast Cancer patient prognoses: a population-based study using the SEER 18 database. PLoS One.

[28] Azim HA, Partridge AH (2014). Biology of breast cancer in young women. Breast Cancer Res.

[29] Swain SM, Nunes R, Yoshizawa C, Rothney M, Sing AP (2015). Quantitative gene expression by recurrence score in ER-positive breast Cancer, by age. Adv Ther.

[30] Hortobagyi GN, Shak S, Sledge GW (2018). Breast cancer-specific mortality in patients with node-negative and node-positive breast cancer guided by the 21-gene assay: a SEER-genomic population-based study.

[31] Liu K-H, Zhang L, Chen J-X, Lian C-L, Wang J, He Z-Y, Wu S-G (2020). Should women with early breast cancer under 40 years of age have a routine 21-gene recurrence score testing: a SEER database study. Breast.

[32] Poorvu PD, Gelber SI, Rosenberg SM, Ruddy KJ, Tamimi RM, Collins LC, Peppercorn J, Schapira L, Borges VF, Come SE, Warner E, Jakubowski DM, Russell C, Winer EP, Partridge AH (2020). Prognostic impact of the 21-gene recurrence score assay among young women with node-negative and node-positive ER-positive/HER2-negative breast Cancer. J Clin Oncol.

[33] Francis PA, Regan MM, Fleming GF, Láng I, Ciruelos E, Bellet M, Bonnefoi HR, Climent MA, Da Prada GA, Burstein HJ (2015). Adjuvant ovarian suppression in premenopausal breast cancer. N Engl J Med.

[34] Francis PA, Pagani O, Fleming GF, Walley BA, Colleoni M, Láng I, Gómez HL, Tondini C, Ciruelos E, Burstein HJ, Bonnefoi HR, Bellet M, Martino S, Geyer CE, Goetz MP, Stearns V, Pinotti G, Puglisi F, Spazzapan S, Climent MA, Pavesi L, Ruhstaller T, Davidson NE, Coleman R, Debled M, Buchholz S, Ingle JN, Winer EP, Maibach R, Rabaglio-Poretti M, Ruepp B, di Leo A, Coates AS, Gelber RD, Goldhirsch A, Regan MM (2018). Tailoring adjuvant endocrine therapy for premenopausal breast Cancer. N Engl J Med.

[35] Gennari R, Curigliano G, Rotmensz N, Robertson C, Colleoni M, Zurrida S, Nolè F, de Braud F, Orlando L, Leonardi MC (2004). Breast carcinoma in elderly women: features of disease presentation, choice of local and systemic treatments compared with younger postmenopasual patients. Cancer.

[36] Davies C, Godwin J, Gray R, Clarke M, Cutter D, Darby S, McGale P, Pan HC, Taylor C, Wang YC (2011). Relevance of breast cancer hormone receptors and other factors to the efficacy of adjuvant tamoxifen: patient-level meta-analysis of randomised trials. Lancet.

[37] Early Breast Cancer Trialists' Collaborative Group (EBCTCG). Effects of chemotherapy and hormonal therapy for early breast cancer on recurrence and 15-year survival: an overview of the randomised trials. Lancet. 2005;365(9472):1687–717. 10.1016/S0140-6736(05)66544-0 15894097

[38] Yu J, Lin C, Huang J, Hong J, Gao W, Zhu S, Wang H, Huang O, Chen X, He J (2021). Abstract PS4-28: efficacy of adjuvant chemotherapy stratified by age and the 21 gene recurrence score in estrogen receptor positive breast cancer. AACR.

