# Supplementary material for: Efficacy of adjuvant chemotherapy stratified by age and the 21-gene recurrence score in estrogen receptor-positive breast cancer
Source: BMC Cancer. 2021 Jun 15;21:707. doi: 10.1186/s12885-021-08461-9 (PMC8207606; doi:10.1186/s12885-021-08461-9)
Supplement: Supplementary file 1 — Additional file 1. [file 12885_2021_8461_MOESM1_ESM.docx]

**Figure S1** Distribution of propensity score before and after matching


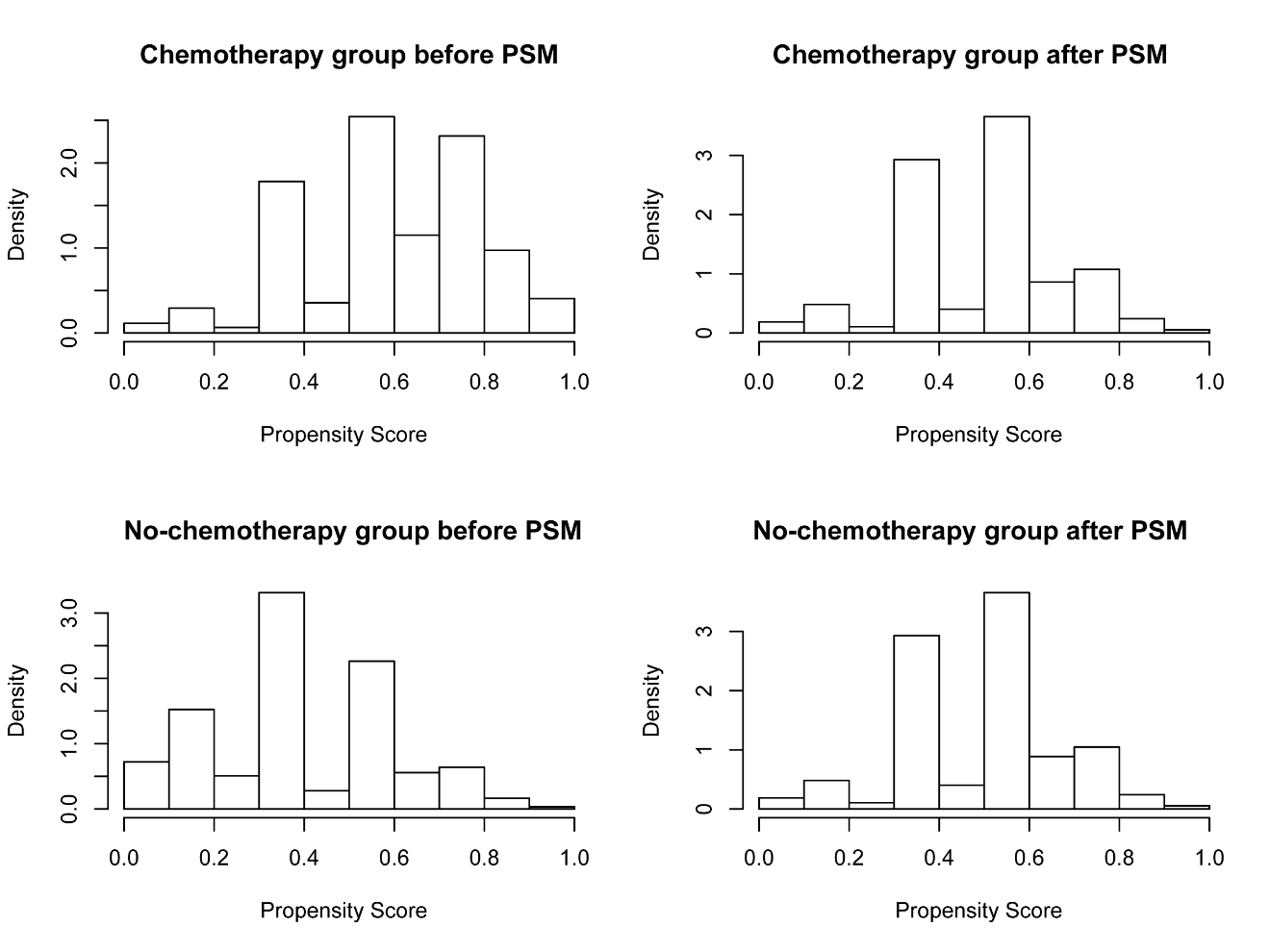


**Figure S2** Interaction between RS and chemotherapy usage for IDFS


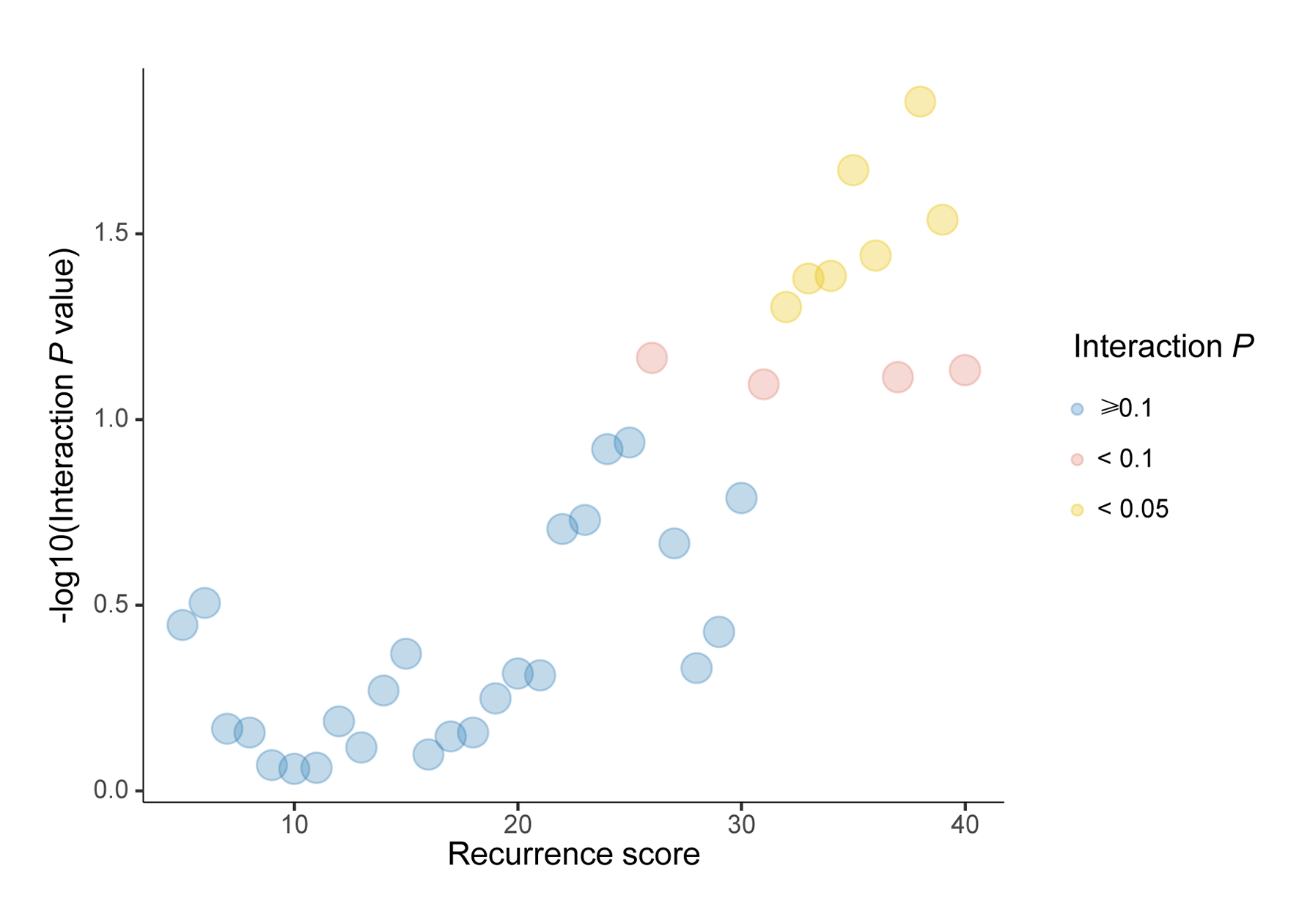


Figure legend: The interaction between chemotherapy usage and RS groups for IDFS was analyzed. Each point in the figure represents the P-value of interaction analysis. Blue points mean no significant interaction was observed (P≥0.1), red points mean the interaction was marginally significant (0.05≤P<0.1), and the yellow points mean the interaction was statistically significant (P<0.05).
